# The mediating role of atrial fibrillation in causal associations between risk factors and stroke: a Mendelian randomization study

**DOI:** 10.4178/epih.e2024005

**Published:** 2023-12-06

**Authors:** Shanmei Qin, Mengmeng Wang, Dipender Gill, Zhizhong Zhang, Xinfeng Liu

**Affiliations:** 1 Department of Neurology, Nanjing Jinling Hospital, Affiliated Hospital of Medical School, Nanjing University, Nanjing, China; 2 Department of Neurology, The First People’s Hospital of Changzhou, The Third Affiliated Hospital of Soochow University, Changzhou, China; 3 Department of Epidemiology and Biostatistics, School of Public Health, St Mary’s Hospital, Imperial College London, London, UK

**Keywords:** Stroke, Mediation, Mendelian randomization, Atrial fibrillation

## Abstract

**OBJECTIVES:**

Atrial fibrillation (AF) contributes to stroke development and progression. We aimed to quantify the mediating role of AF in the causal associations between a wide range of risk factors and stroke via a Mendelian randomization (MR) framework.

**METHODS:**

We assessed the associations of 108 traits with stroke and its subtypes in a 2-sample univariable MR approach, then conducted a bidirectional MR analysis between these 108 traits and AF to evaluate the presence and direction of their causal associations. Finally, to further investigate the extent to which AF mediated the effects of eligible traits on stroke, we applied multivariable and 2-step MR techniques in a mediation analysis where outcomes were restricted to stroke types causally affected by AF (any stroke [AS], any ischemic stroke [AIS], and cardioembolic stroke [CES]).

**RESULTS:**

Among 108 traits, 42 were putatively causal for at least 1 stroke type; of these 42 traits, 20 that had no bidirectional relationship with AF were retained. Finally, 33 associations of 15 eligible traits were examined in the mediation analysis. The mediation analyses for AS, AIS, and CES each included 11 eligible traits. After AF adjustment, the direct effects of all traits on CES were attenuated to null (all p>0.05), while the associations with AS and AIS persisted for most traits (AF-mediated proportion: from 6.6% [95% confidence interval, 2.7 to 0.6] to 52.0% [95% confidence interval, 39.8 to 64.3]).

**CONCLUSIONS:**

The causal associations between all eligible traits and CES were largely mediated through AF, while most traits affected AS and AIS independently of AF.

## INTRODUCTION

Atrial fibrillation (AF), the most common cardiac arrhythmia, is regarded as a specific risk factor for stroke [1]. The prevalence of AF is rising due to the aging population [2]. Strokes related to AF typically result in larger brain infarctions, worse post-stroke outcomes, and higher economic costs than strokes due to other mechanisms [3-5]. Stroke patients with AF have a higher risk of recurrent stroke than those without this arrhythmia [6,7]. Up to 20% of patients experiencing ischemic stroke (IS) have a previous AF diagnosis. However, new-onset AF can be detected in nearly a quarter of patients after a stroke [6], likely due to closer clinical scrutiny, as AF is often paroxysmal and asymptomatic and therefore difficult to capture. Alternatively, stroke itself may cause AF via post-stroke autonomic dysfunction and inflammation [8]. Therefore, the mechanistic relationship between AF and stroke is complex and warrants further investigation.

Mendelian randomization (MR), an epidemiological method that uses genetic variants as instrumental variables (IVs) to determine the causal relationship between an exposure and an outcome, is less susceptible to confounding, reverse causality, and measurement errors that can compromise the validity of observational studies. This is because the genetic variants are randomly assigned at conception [9]. Multivariable Mendelian randomization (MVMR), a recent extension to MR, can be used to investigate mediation [10].

While previous MR studies have reported that AF mediated the effects of thyroid function [11], cardiac traits [12] and some AF-related factors on stroke [13], given multiple shared risk factors and pathways between AF and stroke [14,15], uncertainty remains whether and to what extent AF mediates the causal effects of a wide range of traits on stroke, and whether the mediation effect differs by stroke subtype. We conducted this systematic MR study to assess the causal associations of 108 known or suspected traits with stroke and its subtypes, and to further investigate the mediating role of AF in these associations.

## MATERIALS AND METHODS

### Study design

**Figure 1.**
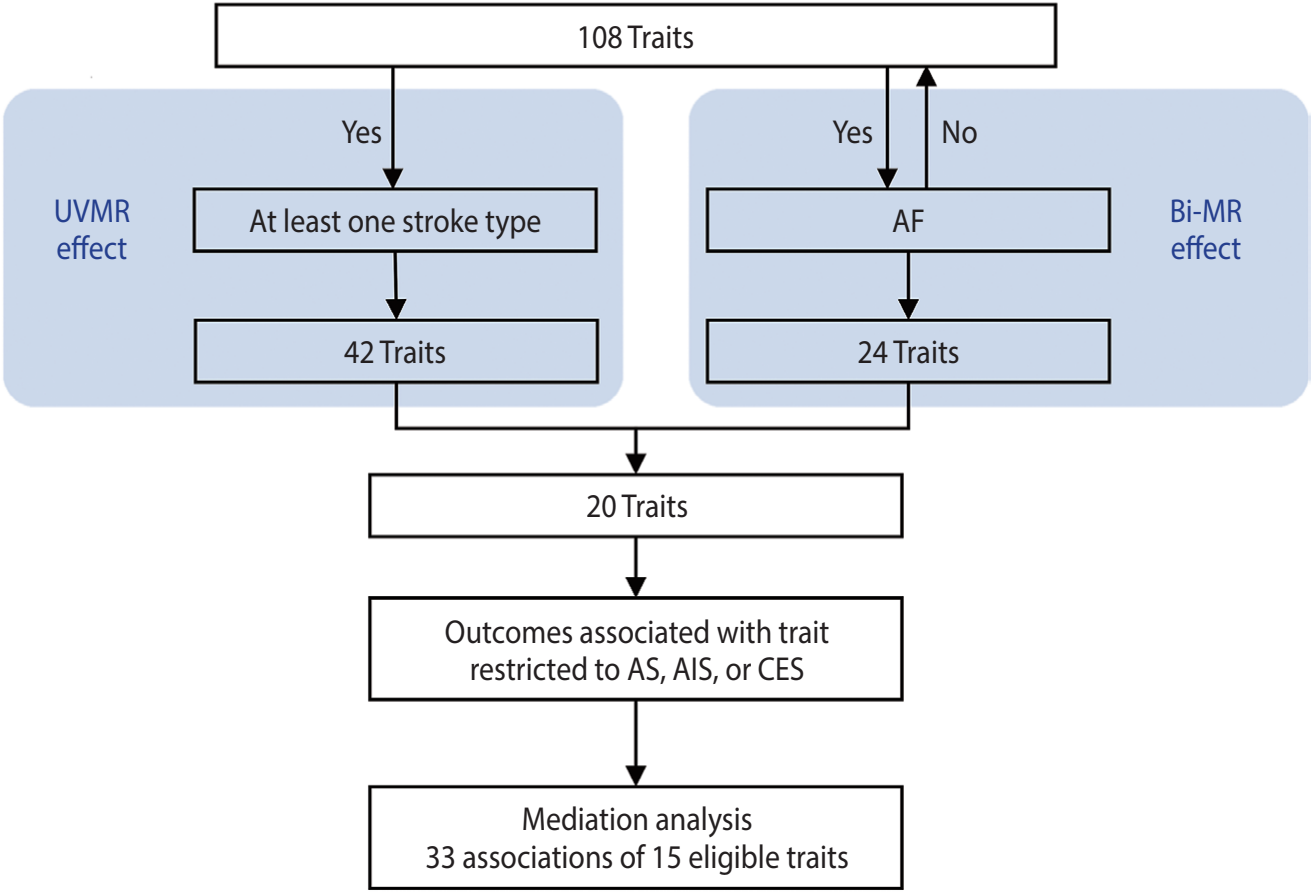
Study design overview. “Effect” indicates whether there was evidence for a causal effect in the Mendelian randomization (MR) analysis (p_FDR_0.05). “Outcomes associated with trait” indicates whether the stroke type associated with the trait was among the stroke types causally affected by AF, namely AS, AIS, and CES. “Mediation analysis” indicates whether the trait was eligible and was further assessed in mediation analysis. UVMR, univariable MR; Bi-MR, bidirectional MR; AF, atrial fibrillation; AS, any stroke; AIS, any ischemic stroke; CES, cardioembolic stroke; FDR, false discovery rate.

**Figure 2.**
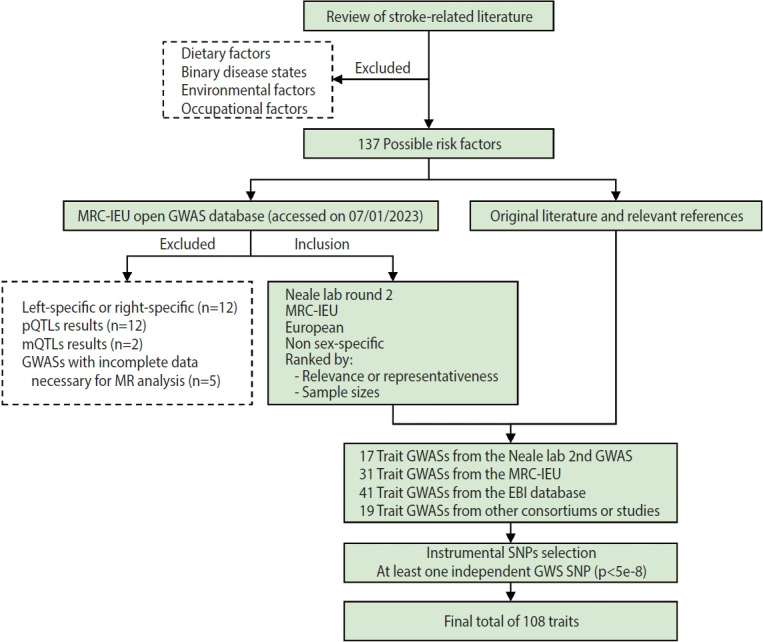
Flow chart illustrating traits selection in the current study. MRC-IEU, Medical Research Council Integrative Epidemiology Unit; GWAS, genome-wide association study; pQTLs, protein quantitative trait loci; mQTLs, metabolite quantitative trait loci; MR, Mendelian randomization; EBI, European Bioinformatics Institute; GWS, genome-wide significant; SNP, single-nucleotide polymorphism.

This study was conducted using several MR approaches, including 2-sample MR [16], bidirectional MR, MVMR [17], and 2-step MR [18], and following the Strengthening the Reporting of Mendelian Randomization Studies guideline [19] (Supplementary Material 1). An overview of the study design is shown in Figure 1. First, we implemented a univariable 2-sample MR study to estimate the associations of all 108 selected traits as well as AF with stroke and it subtypes (see “trait selection” below and Figure 2 for details of the trait selection process). Second, we performed a bidirectional MR analysis between these 108 traits and AF to determine which traits were likely causes, but not consequences, of AF. Subsequently, we conducted a mediation analysis using MVMR and 2-step MR (Supplementary Material 2) to estimate the indirect (i.e., via AF) and direct effect (i.e., independent of AF) of the retained traits on their corresponding stroke outcomes, which were restricted to stroke types that were causally influenced by AF.

### Trait selection and data sources

A flow chart of trait selection in the current study is shown in Figure 2. First, to identify possible stroke risk factors, we consulted the 2022 Stroke Statistics Report from American Heart Association (AHA) [14], recently published systematic reviews of MR studies in stroke [20,21] and relevant articles by searching PubMed. We selected 2 groups of traditional traits: metabolic traits (body mass index [BMI], basal metabolic rate, glycemic traits, blood pressure, blood lipids and lipoproteins, kidney and liver function, etc.) and behavioral traits (smoking, alcohol consumption, and physical activity) except those related to diet (e.g., dietary pattern, specific nutrients). At this stage, binary disease states, as well as environmental and occupational risk factors (e.g., air pollution, lead or noise exposure), were excluded. We also selected non-traditional traits related to anthropometry, lung function, blood cells and hemostasis, hormones, cognitive function and education, early life exposures, sleep, and so forth. In total, 137 possible risk factors were identified. Second, we searched the MRC-IEU Open GWAS database (https://gwas.mrcieu.ac.uk/) in January 2023 for available genome-wide association study (GWAS) summary data that were based on European descendants and were non-sex-specific. At this point we removed traits that were left-specific or right-specific (n=12), traits related to protein (n=12) and metabolite levels (n=2), and traits with incomplete data necessary for MR analysis (n=5), resulting in a total of 17 trait GWASs collected from the Neale lab (second round), 31 from the MRC-IEU, 41 from the European Bioinformatics Institute database and 13 from other consortiums or studies. In addition, 6 other trait GWASs were identified, on height [22]; the estimated glomerular filtration rate based on cystatin C [23] or creatinine [23]; blood urea nitrogen [23]; serum calcium [24]; serum magnesium [25]. In total, 108 traits with at least 1 independent genome-wide significant single-nucleotide polymorphism (SNP, p< 5× 10^-8^) were included in the present study. Details of the GWASs for all traits are provided in Supplementary Material 3.

Summary-level data for AF were obtained from a 2018 GWAS meta-analysis of 60,620 AF cases and 970,216 controls of European ancestry [26], from 6 contributing studies (The Nord-Trøndelag Health Study, deCODE, the Michigan Genomics Initiative, DiscovEHR, UK Biobank, and the AF HRC Consortium). Summary-level data for stroke and its subtypes were available in the MEGASTROKE consortium (European subset) [27], which includes 29 GWASs of any stroke (AS; 40,585 cases/406,111 controls), any ischemic stroke (AIS; 34,217 cases/406,111 controls), and IS subtypes (large-artery atherosclerotic stroke [LAS]: 4,373 cases/146,392 controls; cardioembolic stroke [CES]: 7,193 cases/204,570 controls; small-vessel stroke [SVS]: 5,386 cases/192,662 controls). Stroke subtypes were classified based on the Trial of ORG 10172 in Acute Stroke Treatment (TOAST) criteria [28].

### Genetic instruments

Genetic instruments for the exposure were extracted from the corresponding GWAS by selecting SNPs with genome-wide significance (p<5× 10^-8^), which were further linkage disequilibrium (LD) pruned (R^2^<0.001 within a 10,000 kb window) against the 1,000 Genomes European reference panel to ensure independence. For SNPs in LD, only those with the lowest p-value were retained. SNPs that were not available in the outcome datasets were substituted by proxies with high LD (R^2^ ≥ 0.8). Palindromic SNPs with minor allele frequency above 0.3 are not inferable and were discarded during the harmonization with the outcome data. Further details of the instrumental variable assumptions underlying MR analysis are provided in Supplementary Material 4.

### Univariable Mendelian randomization analysis

We performed summary data 2-sample MR with the random-effects inverse-variance weighted method [29], supplemented by several sensitivity analyses (namely weighted median [30], MR-Egger [31] and MR-Egger intercept tests) to evaluate the robustness of the findings. The weighted median and MR-Egger methods were used to examine estimate consistency, and the I^2^GX statistic was calculated to assess the no measurement error assumption for MR-Egger [32]. The MR-Egger intercept test was used to assess directional pleiotropy. Additionally, Cochran’s Q test was employed to evaluate heterogeneity across IVs [33]. Multiple testing was corrected for in all tests using the Benjamini–Hochberg method to control the false discovery rate (FDR), with a significance threshold set at p_FDR_ < 0.05 [34]. All univariable MR analyses were conducted in R version 4.0.1 (R Foundation for Statistical Computing, Vienna, Austria) with the TwoSampleMR version 0.5.5 [35].

### Mediation analysis

To interrogate the mediating role of AF, we further conducted a mediation analysis using MVMR [17] and 2-step MR [18]. Current MR for mediation analysis assumes a linear exposure-outcome association. The association between AF and stroke was confirmed in univariable Mendelian randomization (UVMR), and 3 stroke types causally influenced by AF were therefore selected as outcomes in the mediation analysis—namely AS, AIS, and CES.

A trait and AF were used as exposures in MVMR, if the trait (1) had an effect on AF; (2) had no bidirectional relationship with AF; and (3) was putatively causal for at least 1 stroke type (Figure 1, Supplementary Material 2). A p_FDR_ < 0.05 was considered as statistically significant. This allowed us to estimate the direct effect of the trait on its corresponding stroke outcome after adjusting for AF. In 2-step MR, we multiplied the causal effect of the trait on AF from the UVMR by the trait-adjusted effect of AF on the stroke outcome from the MVMR to estimate the indirect effect of the trait on stroke via AF. Then, the proportion mediated by AF was estimated by dividing the indirect effect by the total effect. Standard error was derived using the Delta method. MVMR analyses were performed using the TwoSampleMR R package [35]. Conditional F-statistics were calculated to examine instrument strength for each exposure, with F> 10 implying sufficient strength for the analysis [36]. The modified Cochran’s Q-statistics were calculated to quantify heterogeneity [36]. Both the above statistics were obtained using the MVMR R package for MVMR sensitivity analysis [17].

### Ethics statement

All studies had been approved by a relevant ethical review board and all participants had provided informed consent.

## RESULTS

**Figure 3.**
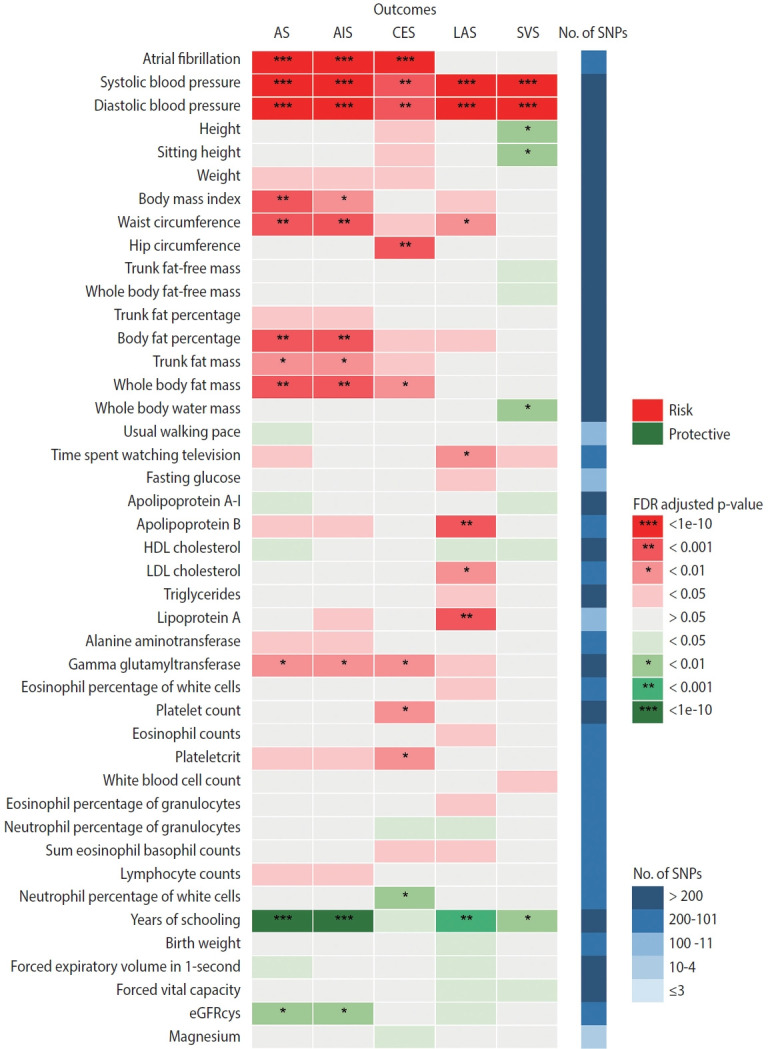
Mendelian randomization (MR) estimates (inverse-variance weighted) for the effects of 42 traits and atrial fibrillation on stroke and its subtypes. Traits presented are those that were causally associated with at least one stroke type. AS, any stroke; AIS, any ischemic stroke; CES, cardioembolic stroke; LAS, large-artery atherosclerotic stroke; SVS, small-vessel stroke; SNP, single-nucleotide polymorphism; FDR, false discovery rate; HDL, high-density lipoprotein; LDL, low-density lipoprotein; eGFRcys, estimated glomerular filtration rate based on cystatin C.

Figure 3 summarizes the causal estimates for the genetic associations of each trait as well as AF with each stroke type obtained from UVMR analysis. Out of all 108 traits, 42 traits were putatively causal for at least 1 stroke type (p_FDR_ < 0.05), of which 21 were associated with AS, 17 with AIS, 18 with CES, 23 with LAS, and 13 with SVS (Figure 3, Supplementary Material 3). Genetic liability to AF was associated with an increased risk of AS (odds ratio [OR], 1.22; 95% confidence interval [CI], 1.18 to 1.27; p_FDR_ = 2.26× 10^-25^ per genetically predicted 1-unit higher log odds of AF), AIS (OR, 1.24; 95% CI, 1.19 to 1.29; p_FDR_ = 1.19× 10^-26^), and CES (OR, 2.02; 95% CI, 1.90 to 2.15; p_FDR_ = 1.40× 10^-111^), but not LAA and SVS (Figure 3, Supplementary Material 3). Sensitivity analyses using alternative MR methods (weighted median and MR-Egger) yielded generally consistent estimates of magnitude and direction (Supplementary Material 3). In the MR-Egger analysis for serum magnesium, the I^2^GX statistic was 0.87 (Supplementary Material 3), which was sufficiently low (less than 0.9), indicating the existence of dilution bias [32]; thus, the results obtained from this method should be interpreted with caution. The MR-Egger intercept test provided evidence for the existence of directional pleiotropy in 42 of 540 associations with stroke (Supplementary Material 3). Heterogeneity in SNP estimates was also observed in most of the exposures (Supplementary Material 3). Notably, insufficient IVs were available for the aforementioned sensitivity analyses of estradiol on stroke (Supplementary Material 3).

**Figure 4.**
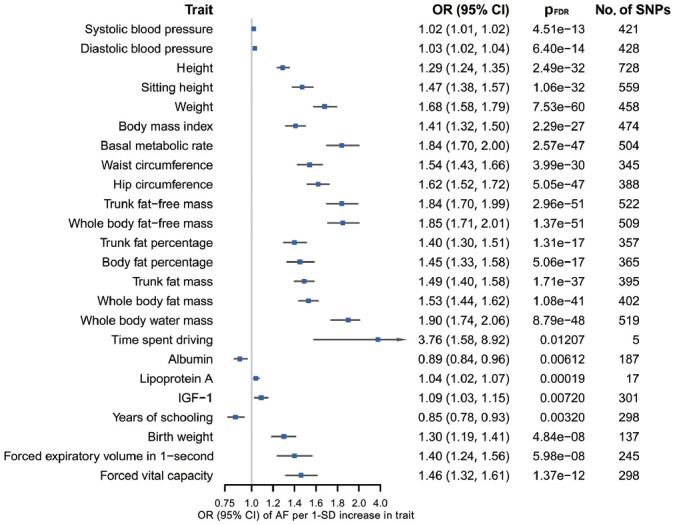
Genetic associations of 24 traits with AF (all p_FDR_0.05). Presented as odds ratios (ORs) with 95% confidence interval (CIs). Based on inverse-variance weighted estimates. The traits presented are those that were causally associated with AF. The ORs are defined as ORs per 1 standard deviation (SD) increase in quantitative traits. AF, atrial fibrillation; pFDR, false discovery rate (FDR) adjusted p-value using the Benjamini–Hochberg method; SNPs, single-nucleotide polymorphisms; IGF, insulin like growth factor.

According to the results of bidirectional MR analysis between AF and all selected traits, 24 traits were found to likely be causes, rather than consequences, of AF (Figure 4, Supplementary Material 3), 20 of which were stroke-associated and were taken forward to the following analyses (Supplementary Material 3). Causal associations of AF with serum calcium and magnesium could not be investigated due to the unavailability of full GWAS summary data. A sensitivity analysis found horizontal pleiotropy in only a few trait-AF and AF-trait associations, despite significant heterogeneity among genetic variants (Supplementary Material 3).

**Figure 5.**
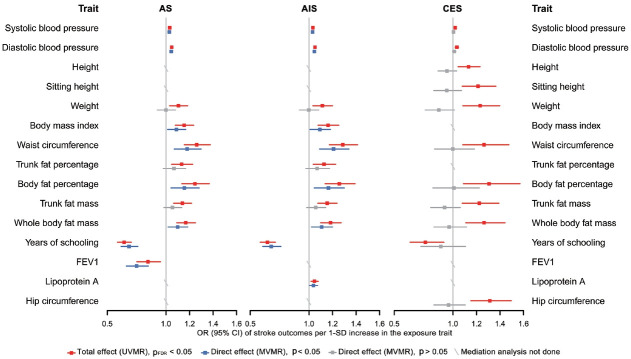
Comparison of the total and direct effects of the eligible traits on corresponding stroke outcomes. Presented as odds ratios (ORs) with 95% confidence interval (CIs). The total effect was obtained from UVMR, and the direct effect from MVMR after adjustment for atrial fibrillation. “Mediation analysis not done” indicates that the trait was not eligible for the mediation analysis of the corresponding stroke outcome. The ORs are defined as ORs per 1 standard deviation (SD) increase of quantitative traits. AS, any stroke; AIS, any ischemic stroke; CES, cardioembolic stroke; FEV1, forced expiratory volume in 1-second; UVMR, univariable Mendelian randomization; MVMR, multivariable Mendelian randomization; pFDR, false discovery rate (FDR) adjusted p-value using the Benjamini–Hochberg method.

Finally, in the mediation analysis where outcomes were restricted to AS, AIS, and CES, 33 associations of 15 retained traits were further assessed using MVMR and 2-step MR (Supplementary Material 3). As shown in Figure 5, mediation analysis for these 3 stroke types each included 11 eligible traits. After adjusting for AF, the causal associations of years of schooling, diastolic and systolic blood pressures (diastolic of blood pressure [DBP] and systolic blood pressure [SBP]) and body fat percentage with CES were attenuated toward the null, with the estimated proportion mediated via AF ranging from 40.7% to 95.2% (Supplementary Material 3). Each of the other 7 traits displayed a negative direct effect on CES risk, opposite to their positive total estimates, but not reaching statistical significance (Figure 5, Supplementary Material 3). Taken together, the vast majority of the effects of all 11 eligible traits on CES risk were mediated through AF (Figure 5).

In the mediation analysis for AS, we observed that the direct and total effects were similar for most traits except weight, trunk fat percentage, and trunk fat mass, and the same was true in the mediation analysis for AIS (Figure 5), indicating that the associations of most traits with AS and AIS persisted after AF adjustment. Furthermore, the mediation analyses for AS and AIS included 10 common traits, and the proportion of the effect of each of these 10 traits explained by AF on AIS was similar to that on AS. Detailed results of mediation analysis are shown in Supplementary Material 3.

For almost all trait-AF pairs, which were analyzed together as the exposures in MVMR, the conditional F-statistics were > 10, except for height-AF, which had a conditional F-statistic of 6 for AF and the results of which should be treated with some caution (Supplementary Material 3). The modified Cochran’s Q-statistic used to evaluate horizontal pleiotropy suggested substantial heterogeneity in most cases, indicative of potential pleiotropy. Detailed results of MVMR sensitivity analysis are available in Supplementary Material 3.

## DISCUSSION

Our findings confirmed the causal associations of a wide range of traits and AF with stroke. Out of all 108 selected traits, 42 had a causal effect on at least 1 stroke type, and 20 of these 42 traits had no bidirectional relationship with AF. We further examined 33 associations of 15 eligible traits in a mediation analysis where outcomes were restricted to AS, AIS, and CES, as stroke types causally affected by AF, and found that after accounting for AF, the direct effects of all 11 traits on CES were attenuated toward the null, while the associations with AS and AIS persisted for most eligible traits except weight, trunk fat percentage and trunk fat mass.

These findings underscore the specific role of AF not only in increasing CES risk, but also in mediating causal effect of risk factors on CES, suggesting the benefit of integrated management of AF in CES prevention. However, the effects of most eligible traits on AS and AIS were found to be independent of their effects on AF. Possible reasons why AF played different mediating roles in CES, AS and AIS are as follows. First, AF may not be as significant a mediator for AS and AIS as it is for CES. Second, the use of composite end-points blurred distinctive changes in the causality of a trait on its corresponding stroke subtypes. In the present study, since AF was not causally associated with LAS and SVS, it was less likely that AF would cause material changes in the relationship between traits and these 2 stroke subtypes, but at the same time, AF adjustment attenuated the associations between all eligible traits and CES to null. That is, the direct effects of traits on AIS independent of AF may actually be attributable to their effects on LAS or SVS.

There are also reasons why we did not find mediating effects involving AF for several traits, especially in their associations with AS and AIS. A trait might affect a certain component of susceptibility to AF, while the affected part has little causal effect on stroke risk. Alternatively, although AF shares many risk factors or pathways with stroke, their relative importance differs. For example, high blood pressure is recognized as the most potent risk factor for stroke [38], supporting our findings that almost all the causal associations of blood pressure with stroke (except SBP-CES, p=0.240; DBPCES, p=0.129; Figure 5, Supplementary Material 3) were independent of AF. Additionally, years of schooling was the only common protective factor for all 3 stroke types in the mediation analysis, and its negative effects on stroke risk may be related to reduced BMI, SBP, and smoking behavior [38]. Collectively, our results support that aggressive risk factor modification remains paramount for AF patients, especially the control of blood pressure and obesity-related factors, which could limit the development of AS and any AIS. All the above-stated findings indicate that the causal effects of all selected traits on stroke, as well as whether and to what extent AF mediates these effects, may vary among different stroke types, stressing the need for distinguishing stroke types in clinical practice and trials.

Our study has some limitations. This was not a phenome-wide MR analysis, but a study limited to possible risk factors selected according to published studies focusing on stroke. In addition, although 108 traits were included in our study, we may have still missed some due to the unavailability of full GWAS summary data from the public repository or domain. Since our mediation analysis restricted the outcomes to AS, AIS, and CES, and some traits were excluded on account of their bidirectional relationship with AF, studies with a broader range of traits are warranted, as well as further research on potential mediators between traits and other stroke types (e.g., LAA, SVS, and intracerebral hemorrhage). Genetic data related to stroke severity (e.g., brain infarct size and location), poststroke outcomes (e.g., National Institutes of Health Stroke Scale) or AF subtypes are also urgently needed to unravel the pathophysiology of stroke and AF and develop novel preventive, therapeutic, and rehabilitation strategies. The traditional MR analysis applied in this study often presumes linearity, while ignoring the possible non-linear relationship between an exposure and an outcome [39]; therefore, potential non-linear associations should be further considered and evaluated. To obtain valid MR results, 3 core assumptions must be satisfied and 2-step MR used in mediation analysis further requires no exposure-mediator interactions [9]. Except for the “relevance” assumption, the other 2 assumptions are difficult to assess and verify [40]. However, several sensitivity analyses were performed to address this issue. There was sample overlap between exposure and outcome GWASs in several analyses, which may have induced potential overfitting and bias in the MR estimates [41]; for example, there was around 38% sample overlap between the UK Biobank and AF GWAS study. Evidence of weak instrument bias may still be present, as indicated by a conditional F-statistic of 6 for AF when analyzed together with height as exposures in MVMR. Moreover, the limited number of genetic instruments for several traits, especially estradiol, reduced the robustness and precision of our MR analysis findings. The use of log odds of the binary mediator (AF) and outcomes (stroke and its subtypes) would introduce bias into MR analysis due to the non-collapsibility of ORs [42]. Finally, this study was conducted using summary data from individuals of European ancestry, limiting the generalizability of our findings to other ancestries.

In conclusion, we found evidence that the causal associations between all eligible traits and CES were largely mediated through AF, while most traits affected the risk of AS and AIS independently of AF. These findings suggest that integrated management of AF will be beneficial for preventing stroke, especially CES. Meanwhile, interventions targeting some key risk factors beyond AF, such as high blood pressure and obesity-related factors, remain essential to limit the development of AS and AIS. The mediating role of AF varied significantly among stroke types, highlighting the need for accurate disease classification in clinical practice and trials.

## Data Availability

MRC-IEU UK Biobank GWAS database can be publicly available at https://gwas.mrcieu.ac.uk/. All data generated or analyzed during this study can be found in the cited references and supplementary files.

## Supplementary materials

**Supplementary Material 1** STROBE-MR checklist **Supplementary Material 2** Supplementary Fig. S1 Two-step Mendelian randomization used for mediation analysis. **Supplementary Material 3** Supplementary Tables S1-S11 are provided in a separate Excel file. **Supplementary Material 4** Supplementary Method S1: Mendelian randomization analysis assumptions.
**Supplementary References**
