# Supplementary material for: The mediating role of atrial fibrillation in causal associations between risk factors and stroke: a Mendelian randomization study
Source: Epidemiol Health. 2023 Dec 6;46:e2024005. doi: 10.4178/epih.e2024005 (PMC11040217; doi:10.4178/epih.e2024005)
Supplement: Supplementary file 1 [file epih-46-e2024005-Supplementary.pdf]

SUPPLEMENTARY MATERIAL

OUTLINE

- **Supplementary Material 1……………………………………………………………2**

  STROBE-MR checklist

- **Supplementary Material 2……………………………………………………………7**

  Supplementary Fig. S1 Two-step Mendelian randomization used for mediation analysis.

- **Supplementary Material 3……………………………………………………………8**

  Supplementary Tables S1-S11 are provided in a separate Excel file.

- **Supplementary Material 4……...…………………………………………………11**

  Supplementary Method S1: Mendelian randomization analysis assumptions.

- **Supplementary References……………...……………………………………………12**

Supplementary Material 1

STROBE-MR checklist [1]

Item No.	Section		Checklist item	Page No.	Relevant text from manuscript
1	**TITLE and ABSTRACT**		Indicate Mendelian randomization (MR) as the study's design in the title and/or the abstract if that is a main purpose of the study	1	Title and the abstract section
	**INTRODUCTION**				
2	**Background**		Explain the scientific background and rationale for the reported study. What is the exposure? Is a potential causal relationship between exposure and outcome plausible? Justify why MR is a helpful method to address the study question	2	Introduction (paragraphs 1-2)
3	**Objectives**		State specific objectives clearly, including pre-specified causal hypotheses (if any). State that MR is a method that, under specific assumptions, intends to estimate causal effects	2	Introduction (paragraph 3)
	**METHODS**				
4	**Study design and data sources**		Present key elements of the study design early in the article. Consider including a table listing sources of data for all phases of the study. For each data source contributing to the analysis, describe the following:		
		a)	Setting: Describe the study design and the underlying population, if possible. Describe the setting, locations, and relevant dates, including periods of recruitment, exposure, follow-up, and data collection, when available.	3-4	"Trait selection and Data sources" section within Methods (paragraphs 2-3), Table S1
		b)	Participants: Give the eligibility criteria, and the sources and methods of selection of participants. Report the sample size, and whether any power or sample size calculations were carried out prior to the main analysis	3-4	"Trait selection and Data sources" section within Methods (paragraphs 2-3)
		c)	Describe measurement, quality control and selection of genetic variants	3-4	"Trait selection and Data sources" section within Methods (paragraphs 2-3)

		d)	For each exposure, outcome, and other relevant variables, describe methods of assessment and diagnostic criteria for diseases	3-4	"Trait selection and Data sources" section within Methods (paragraphs 2-3)
		e)	Provide details of ethics committee approval and participant informed consent, if relevant		N/A
5	**Assumptions**		Explicitly state the three core IV assumptions for the main analysis (relevance, independence and exclusion restriction) as well assumptions for any additional or sensitivity analysis	5	“Univariable Mendelian randomization analysis” section within Methods (paragraph 5)
6	**Statistical methods: main analysis**		Describe statistical methods and statistics used		
		a)	Describe how quantitative variables were handled in the analyses (i.e., scale, units, model)	3-4	Methods (paragraphs 2-3), Table S1
		b)	Describe how genetic variants were handled in the analyses and, if applicable, how their weights were selected	5	“Genetic instruments” section within Methods (paragraph 4)
		c)	Describe the MR estimator (e.g. two-stage least squares, Wald ratio) and related statistics. Detail the included covariates and, in case of two-sample MR, whether the same covariate set was used for adjustment in the two samples	5-6	Methods (paragraphs 5-7)
		d)	Explain how missing data were addressed	5	“Genetic instruments” section within Methods (paragraph 4)
		e)	If applicable, indicate how multiple testing was addressed	5	“Univariable Mendelian randomization analysis” section within Methods (paragraph 5)
7	**Assessment of assumptions**		Describe any methods or prior knowledge used to assess the assumptions or justify their validity	5-6	Methods (paragraphs 5-7)
8	**Sensitivity analyses and additional analyses**		Describe any sensitivity analyses or additional analyses performed (e.g. comparison of effect estimates from different approaches, independent replication, bias analytic techniques, validation of instruments, simulations)	5-6	Methods (paragraphs 5 and 7)
9	**Software and pre-registration**				

		a)	Name statistical software and package(s), including version and settings used	5-6	Methods (paragraphs 5 and 7)
		b)	State whether the study protocol and details were pre-registered (as well as when and where)		N/A
	**RESULTS**				
10	**Descriptive data**				
		a)	Report the numbers of individuals at each stage of included studies and reasons for exclusion. Consider use of a flow diagram		N/A
		b)	Report summary statistics for phenotypic exposure(s), outcome(s), and other relevant variables (e.g. means, SDs, proportions)		Table S1
		c)	If the data sources include meta-analyses of previous studies, provide the assessments of heterogeneity across these studies		N/A
		d)	For two-sample MR:		
			i. Provide justification of the similarity of the genetic variant-exposure associations between the exposure and outcome samples	3-4	Methods (paragraphs 2-3)
			ii. Provide information on the number of individuals who overlap between the exposure and outcome studies	10	Discission (paragraph 4)
11	**Main results**				
		a)	Report the associations between genetic variant and exposure, and between genetic variant and outcome, preferably on an interpretable scale		N/A
		b)	Report MR estimates of the relationship between exposure and outcome, and the measures of uncertainty from the MR analysis, on an interpretable scale, such as odds ratio or relative risk per SD difference	6-8	Results (paragraphs 1-4), Tables S2-S4, S10-S11
		c)	If relevant, consider translating estimates of relative risk into absolute risk for a meaningful time period		N/A
		d)	Consider plots to visualize results (e.g. forest plot, scatterplot of associations between genetic variants and outcome versus between genetic variants and exposure)		Figures 3-5

12	**Assessment of assumptions**				
		a)	Report the assessment of the validity of the assumptions	6-7	Results (paragraphs 1-2)
		b)	Report any additional statistics (e.g., assessments of heterogeneity across genetic variants, such as I^2, Q statistic or E-value)	6-8	Results (paragraphs 1-2, 5), Tables S2-S4, S7-S10
13	**Sensitivity analyses and additional analyses**				
		a)	Report any sensitivity analyses to assess the robustness of the main results to violations of the assumptions	6-8	Results (paragraphs 1-2, 5), Tables S7-S10
		b)	Report results from other sensitivity analyses or additional analyses	6-8	Results (paragraphs 1-2, 5), Tables S7-S10
		c)	Report any assessment of direction of causal relationship (e.g., bidirectional MR)	6-8	Results (paragraphs 1-2, 5), Tables S7-S10
		d)	When relevant, report and compare with estimates from non-MR analyses		N/A
		e)	Consider additional plots to visualize results (e.g., leave-one-out analyses)		N/A
	**DISCUSSION**				
14	**Key results**		Summarize key results with reference to study objectives	8	Discussion (paragraph 1)
15	**Limitations**		Discuss limitations of the study, taking into account the validity of the IV assumptions, other sources of potential bias, and imprecision. Discuss both direction and magnitude of any potential bias and any efforts to address them	10	Discussion (paragraph 4)
16	**Interpretation**				
		a)	Meaning: Give a cautious overall interpretation of results in the context of their limitations and in comparison with other studies	8-9	Discussion (paragraphs 2-3)
		b)	Mechanism: Discuss underlying biological mechanisms that could drive a potential causal relationship between the investigated exposure and the outcome, and whether the gene-environment equivalence assumption is	8-9	Discussion (paragraphs 2-3)

		reasonable. Use causal language carefully, clarifying that IV estimates may provide causal effects only under certain assumptions		
	c)	Clinical relevance: Discuss whether the results have clinical or public policy relevance, and to what extent they inform effect sizes of possible interventions	8-11	Discussion (paragraphs 2, 3, 5)
17	**Generalizability**	Discuss the generalizability of the study results (a) to other populations, (b) across other exposure periods/timings, and (c) across other levels of exposure	11	Discussion (paragraph 4)
	**OTHER INFORMATION**			
18	**Funding**	Describe sources of funding and the role of funders in the present study and, if applicable, sources of funding for the databases and original study or studies on which the present study is based		N/A
19	**Data and data sharing**	Provide the data used to perform all analyses or report where and how the data can be accessed, and reference these sources in the article. Provide the statistical code needed to reproduce the results in the article, or report whether the code is publicly accessible and if so, where	3-6	Methods (paragraphs 2, 3, 5, 7)
20	**Conflicts of Interest**	All authors should declare all potential conflicts of interest	11	“Conflict of Interest” section

Supplementary Material 2

SUPPLEMENTARY FIGURE

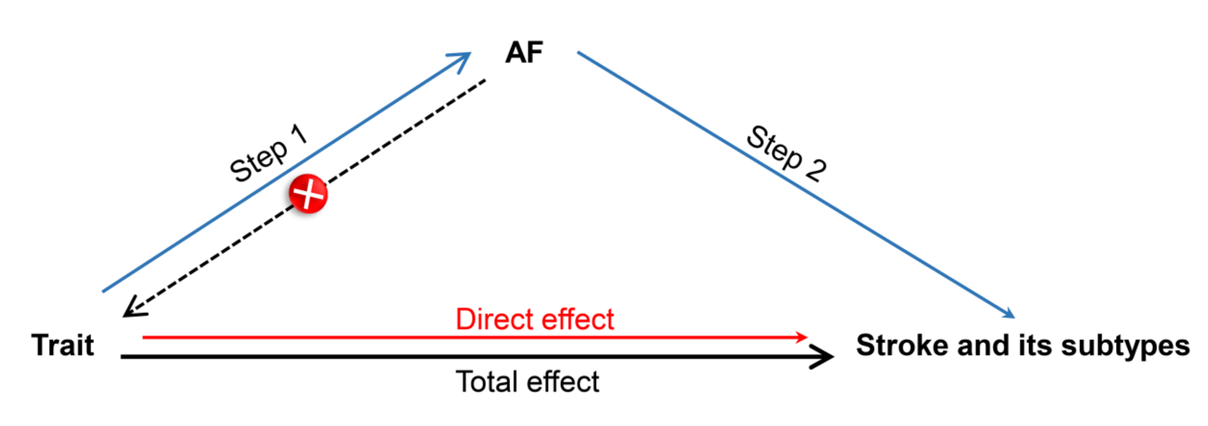

Fig. S1 Two-step Mendelian randomization used for mediation analysis.

Step 1: the effect of the trait on AF, step 2: the trait-adjusted effect of AF on the stroke risk. “Total effect” indicates the effect of the trait on stroke, and “Direct effect” indicates the effect of the trait on stroke independent of AF. The red cross indicates that eligible traits for mediation analysis had no bidirectional relationship with AF. AF, atrial fibrillation.

Supplementary Material 3

SUPPLEMENTARY TABLES

Table S1. GWAS summary data information of 108 selected traits in this analysis.

Table S2. MR estimates from all selected traits and AF to stroke outcomes, presented as OR with 95% CI.
nsnp: number of independent single-nucleotide polymorphisms; or: odds ratio per one standard deviation (SD) increase of quantitative traits or per genetically predicted 1-unit higher log odds of AF; or_lci95 and or_uci95: lower and upper boundary of 95% confidence interval (CI); Isq: I^2GX statistic calculated to assess the no measurement error assumption for MR-Egger.

Table S3. MR estimates from all selected traits to AF, presented as OR with 95% CI.
nsnp: number of independent single-nucleotide polymorphisms; or: odds ratio per one standard deviation (SD) increase of quantitative traits; or_lci95 and or_uci95: lower and upper boundary of 95% confidence interval (CI); Isq: I2GX statistic calculated to assess the no measurement error assumption for MR-Egger.

Table S4. MR estimates from AF to all selected traits.
Except serum calcium and magnesium due to the unavailability of full GWAS summary data. Results are presented as beta coefficients with 95% confidence interval (CI) per genetically predicted 1-unit higher log odds of AF; nsnp: number of independent single-nucleotide polymorphisms; lo_ci and up_ci: lower and upper boundary of 95% confidence interval; Isq: I2GX statistic calculated to assess the no measurement error assumption for MR-Egger.

Table S5. Multiple-testing correction for 545 associations of 108 selected traits and AF with stroke and its subtypes.
Based on inverse-variance weighted (IVW) estimates, except oestradiol (whose estimates are based on a single Wald ratio); PFDR: false discovery rate (FDR) adjusted P-value using Benjamini–Hochberg method; evidence: whether PFDR is less than 0.05.

Table S6. Multiple-testing correction for bidirectional associations between 108 selected traits and AF.

Based on inverse-variance weighted (IVW) estimates, except oestradiol (whose estimates are based on a single Wald ratio); trait_AF_: MR estimates from all selected traits to AF; AF_trait_: MR estimates from AF to all selected traits except serum calcium and magnesium; PFDR: false discovery rate (FDR) adjusted P-value using Benjamini–Hochberg method; evidence: whether PFDR is less than 0.05.

Table S7. MR sensitivity analysis for the associations of all selected traits and AF with stroke.

Except oestradiol due to the lack of enough IVs available. Cochran's Q-statistic and P-value were used to assess heterogeneity. MR-Egger intercept tests were used to assess directional pleiotropy.

Table S8. MR sensitivity analysis for the associations of all selected traits with AF.

Except oestradiol due to the lack of enough IVs available. Cochran's Q-statistic and P-value were used to assess heterogeneity. MR-Egger intercept tests were used to assess directional pleiotropy.

Table S9. MR sensitivity analysis for the associations of AF with all selected traits.

Except serum calcium and magnesium due to the unavailability of full GWAS summary data. Cochran's Q-statistic and P-value were used to assess heterogeneity. MR-Egger intercept tests were used to assess directional pleiotropy.

Table S10. MVMR results for the effects of eligible traits on stroke outcomes.

Along with MVMR sensitivity analysis results. Estimated after adjusting for atrial fibrillation (AF). Presented as OR with 95% CI. nsnp: number of independent single-nucleotide polymorphisms; or: odds ratio per one standard deviation (SD) increase of quantitative traits or per genetically predicted 1-unit higher log odds of AF; or_lci95 and or_uci95: lower and upper boundary of 95% confidence interval (CI). Conditional F-statistics (condF) were calculated to examine instrument strength for each exposure. The modified Cochran's Q statistics (Qstat) were calculated to quantify heterogeneity.

Table S11. Mediation analysis of the effects of eligible traits on stroke outcomes.

Indirect effect of each trait was calculated using product of coefficients method. Standard

error (SE) was derived using the Delta method. “Direct effect” indicates the effect of eligible traits on stroke outcomes after adjustment for AF, “Mediation effect” indicates the effect of eligible traits on stroke outcomes via AF and “Total effect” indicates the effect of eligible traits on stroke outcomes. Estimates for “Total effect” and “effect of eligible traits on AF” were obtained by using inverse-variance weighted (IVW). AF: atrial fibrillation; AS: any stroke; AIS: any ischemic stroke; CES: cardioembolic stroke. * The upper boundary of 95% confidence interval (CI) for the proportion of mediation effect is no more than 100%.

Supplementary Material 4

SUPPLEMENTARY METHOD

Method S1: Mendelian randomization analysis assumptions.

MR is based on three instrumental variable assumptions: (1) genetic instruments must be associated with the exposure of interest (relevance), (2) genetic instruments must be not associated with any confounder between the exposure and outcome (independence), and (3) genetic instruments do not affect the outcome except via their association with the exposure (exclusion restriction) [1].

The ‘relevance’ assumption is satisfied by defining instruments using the genome-wide significant ($P < 5 \times 10^{-8}$) SNPs from the corresponding GWASs. To assess potential violation of the second and third assumptions, we performed several sensitivity analyses with different assumptions, namely weighted median [2], MR-Egger [3] and MR-Egger intercept tests.

SUPPLEMENTARY REFERENCES

1. Skrivankova VW, Richmond RC, Woolf BAR, et al. Strengthening the Reporting of Observational Studies in Epidemiology Using Mendelian Randomization: The STROBE-MR Statement. JAMA. 2021;326(16):1614-1621.
2. Bowden J, Davey Smith G, Haycock PC, Burgess S. Consistent Estimation in Mendelian Randomization with Some Invalid Instruments Using a Weighted Median Estimator. Genet Epidemiol. 2016;40(4):304-314.
3. Bowden J, Davey Smith G, Burgess S. Mendelian randomization with invalid instruments: effect estimation and bias detection through Egger regression. Int J Epidemiol. 2015;44(2):512-525.
